# AIM2 Drives Joint Inflammation in a Self-DNA Triggered Model of Chronic Polyarthritis

**DOI:** 10.1371/journal.pone.0131702

**Published:** 2015-06-26

**Authors:** Christopher Jakobs, Sven Perner, Veit Hornung

**Affiliations:** 1 Institute of Molecular Medicine, University Hospital Bonn, University of Bonn, Bonn, Germany; 2 Department of Prostate Cancer Research, Institute of Pathology, Center for Integrated Oncology Köln/Bonn, University Hospital Bonn, Bonn, Germany; French National Centre for Scientific Research, FRANCE

## Abstract

Mice lacking DNase II display a polyarthritis-like disease phenotype that is driven by translocation of self-DNA into the cytoplasm of phagocytic cells, where it is sensed by pattern recognition receptors. While pro-inflammatory gene expression is non-redundantly linked to the presence of STING in these mice, the contribution of the inflammasome pathway has not been explored. To this end, we studied the role of the DNA-sensing inflammasome receptor AIM2 in this self-DNA driven disease model. Arthritis-prone mice lacking AIM2 displayed strongly decreased signs of joint inflammation and associated histopathological findings. This was paralleled with a reduction of caspase-1 activation and pro-inflammatory cytokine production in diseased joints. Interestingly, systemic signs of inflammation that are associated with the lack of DNase II were not dependent on AIM2. Taken together, these data suggest a tissue-specific role for the AIM2 inflammasome as a sensor for endogenous DNA species in the course of a ligand-dependent autoinflammatory condition.

## Introduction

The innate immune system has evolved a conserved set of so-called pattern recognition receptors (PRRs) to sense the presence of microbial pathogens. PRRs detect microbe-associated molecular patterns (MAMPs) as non-self and initiate signaling cascades geared at eliminating the microbial threat [1]. Under certain circumstances, these receptors can also respond to self-derived molecules. This, for example, includes endogenous nucleic acids that have gained access to compartments that are usually devoid of these [2]. Accidental activation of compartmentalized PRRs by endogenous nucleic acids is additionally prevented by the presence of nucleases that degrade and thereby deplete potential self-ligands under steady state conditions. These nucleases are expressed in a compartment and cell-type specific manner, creating a non-redundant system of nuclease activity, which prevents accidental activation of nucleic acid sensing PRRs. The importance of this safeguard system is impressively documented by the fact that defects in a number of these nucleases can result in severe sterile inflammatory conditions that are triggered by nucleic acid sensing PRRs [3]. Aicardi–Goutières syndrome (AGS), for example, is a rare genetic inflammatory disorder, in which defects in nucleic acid degrading or metabolizing enzymes can result in the spontaneous production of antiviral cytokines in cerebrospinal fluid and serum, manifesting as a sub-acute encephalopathy [4, 5].

Detection of cytosolic DNA triggers at least two distinct core signaling cascades: On the one hand, cytosolic DNA leads to the activation of the nucleotidyltransferase cGAS, which upon DNA binding produces a 2’-5’ linked cyclic dinucleotide second messenger molecule that in turn binds to and activates the ER-resident receptor STING. STING activation results in its translocation to a perinuclear Golgi compartment, where it achieves its signaling competent state, culminating in the activation of antiviral and pro-inflammatory gene expression [6, 7]. While these two components form the non-redundant core of cytosolic DNA mediated antiviral gene expression, additional factors have been described to function in concert with cGAS and or STING [8]. In myeloid cells, cytosolic DNA is additionally sensed by the PYHIN protein AIM2, which forms an ASC-dependent inflammasome complex upon ligand binding. Inflammasome activation results in the processing and activation of pro-caspase-1, which itself leads to the processing of pro-cytokines such as IL-1β and IL-18 [9–12]. At the same time, inflammasome activation leads to the induction of a myeloid cell specific cell death, known as pyroptosis [13]. AIM2 has shown to be involved in the recognition of a number of microbial pathogens, such as DNA viruses or bacteria that release microbial DNA into the cytoplasm during their life cycle. However, a role for AIM2 in the recognition of endogenous self-DNA has not been established so far [14].

To study the possible role of AIM2 in the context of a self-DNA driven autoinflammatory disease, we made use of a DNase II-deficiency mouse model [15]. In this mouse model, absence of the lysosomal endonuclease DNase II (*Dnase2*) leads to a defect in disposing of DNA in lysosomal compartments [16]. This subsequently results in the translocation of undigested DNA into the cytoplasm, which in turn results in the activation of STING leading to unabated production of type I IFNs and pro-inflammatory cytokines [17]. Spontaneous cytokine production is already seen in early fetal development of *Dnase2*-deficient mice, due to the fact that macrophages play an important role in disposing of expelled nuclei from erythroid precursor cells in so-called erythroblastic islands in the fetal liver or bone marrow in the course of definitive erythropoiesis [16, 18]. This macrophage-dependent type I IFN production in the context of *Dnase2*-deficiency results in lethal anemia, which can be fully rescued by ablating type I IFN production or its activity (e.g. *Ifnar1* ^-/-^) in these mice [19]. While *Dnase2*-*Ifnar1* DKO mice are born healthy, macrophages in these mice are still responding to undigested DNA by the up regulation of pro-inflammatory genes. In fact, 3–6 months after birth *Dnase2* ^-/-^ *Ifnar1* ^-/-^ mice develop a systemic auto-inflammatory disease, most prominently affecting the joints with chronic polyarthritis mimicking rheumatoid arthritis in humans [15]. Even though accompanied by the production of autoantibodies, this chronic polyarthritis is independent of the adaptive branch of the immune system, yet rather driven by the unabated production of pro-inflammatory cytokines, most prominently TNF, IL-6 and IL-1β [20]. In fact, in diseased joints of *DNase2*-deficient animals these cytokines appear to regulate each other’s expression in a mutually dependent fashion. To this effect, blocking the activity of one of these cytokines blunts the expression of the others and thereby greatly ameliorates disease progression [20]. Il-18, another potential candidate cytokine that has been associated with the development and progression of arthritis, is not involved in this disease model. In fact, IL-18^-/-^ mice still develop polyarthritis and express pro-inflammatory cytokines in the context of *Dnase2*-deficiency [20].

The inflammatory response in the context of defective disposal of erythroid precursors by macrophages in erythroblastic islands is an example of how exogenous self-DNA triggers the activation of innate sensing pathways in phagocytic cells. A recent study, however, has demonstrated that *DNase2*-deficiency also triggers cell-autonomous immune responses in cells that are not phagocytic [21]. In fact, these studies show that damaged DNA expelled from nuclei is normally subjected to DNase II mediated degradation, involving a process that depends on nuclear export and subsequent autophagy-dependent delivery to lysosomal structures. As such, next to its function in degrading apoptotic material from other cells, DNase II prevents the initiation of a cell autonomous innate immune response by disposing of nuclear-derived DNA.

Given the overlapping ligand spectrum of the cGAS-STING axis as well as the inflammasome sensor AIM2, we hypothesized that AIM2 might also be activated by the presence of cytosolic DNA in the course of *Dnase2*-deficiency. Moreover, we speculated that part of the disease activity in this mouse model could indeed be attributed to AIM2.

## Materials and Methods

### Mice

Mice deficient for *Dnase2* and *Ifnar1* on a C57BL/6 background were previously described [15]. *Aim2* ^*-/-*^ mice originate from crossing of PGK-Cre [22] mice with *Aim2* ^*flox/flox*^ mice, which were generated by Taconic Artemis on a C57BL/6 background (S1 Fig). Following the outbreeding of PGK-Cre, the genetic background of *Aim2* ^*-/-*^ mice was validated to be 99.23% C57BL/6J using SNP genotyping. Subsequently, *Aim2* ^*-/-*^ mice were backcrossed with C57BL/6J for another 6 generations. Primers used for genotyping of *Aim2* deficient mice: *AF*: 5’- TTGAAGAGATGGGACAGCAA-3’; *A1*: 5’-TGAACTTCCAGGACACAAAG-3’; *A2*: 5’-GCAAGCAGTTAACATTTTGAAGC-3’. All described strains were housed under specific pathogen-free conditions. Studies are approved by the district government of Northrhine Westphalia, here the “State Office for protection of nature, environment and consumers” (LANUV Landesamt für Natur, Umwelt und Verbraucherschutz, Leibnizstraße 10, 45659 Recklinghausen, Germany), which is the responsible agency. The application number is: 84.02.04.2014.A436. Animal experiments and handling were also supervised by Institutional Animal Care and Use Committee (IACUC) of the medical faculty Bonn (HET, House of Experimental Therapy, Sigmund-Freud Str. 25, 53127 Bonn, Germany). Mice did not show any signs of pain, distress or motor dysfunction. Mice were euthanized according to the FELASA guidelines by cervical dislocation at the respective time points indicated.

### Clinical score

Swelling of the fore- and hind pads was analyzed in a blinded fashion, in monthly intervals and scored as follows: 0, no swelling; 1, mild swelling; 2, severe swelling. The scores were summed, and a total score (maximum 8) was assigned to each mouse [15].

### Histopathology

Hind limbs of sacrificed mice were skinned and fixed in 4% paraformaldehyde solution, followed by a decalcification step in 20% EDTA (m/v, pH 7,0) at room temperature, under rotation, for 10 d, with daily buffer change. Next samples became embedded in paraffin and sections of 4 μm were generated. HE stains were performed manually using Haematoxylin and Eosin solutions. For immunohistochemistry analysis paraffin-embedded sections were stained using a Ventana Benchmark XT system (Ventana Medical Systems, Tucson, AZ) together with primary antibody rabbit monoclonal to MMP3 (ab52915; dilution 1:300; Abcam, Cambridge, UK), Rat monoclonal to F4/80 (ab6640; dilution 1:500; Abcam, Cambridge, UK) and with secondary antibody Rabbit Anti-Rat IgG (ab7099; 1:1000; Abcam, Cambridge, UK). HE, F4/80 and MMP3 stains were scored as follows: 0, no overt signs of synovitis / infiltration; 1, low grade synovitis / infiltration; 2, intermediate grade synovitis / infiltration; 3, high grade synovitis / infiltration.

### RNA and protein isolation

Limbs of sacrificed mice were skinned and ground in liquid nitrogen and either transferred to TRIzol (Life technologies; Carlsbad, CA) for mRNA isolation or to tissue lysis buffer (T-PER, Tissue Protein Extraction Reagent, Pierce combined with Protease Inhibitor Tablets; Pierce/Thermo Scientific; Pittsburgh, PA) for protein extraction.

### Immunoblotting

Protein samples were diluted in 2x Laemmli buffer and boiled at 95°C for 5 min. Samples were separated by SDS gel electrophoresis for 2 h and further transferred to a 0.2 μM Nitrocellulose membrane [23]. As indicated, blots were incubated with rabbit polyclonal antibody to anti caspase-1 p10 (sc-514; Santa Cruz Biotechnology; Santa Cruz, LA), rabbit monoclonal anti MMP3 (ab52915; Abcam; Cambridge, UK) and mouse monoclonal anti Viperin (MABF106; Merck Millipore; Billerica, MA). Membranes were developed using ECL Western blotting substrate (Pierce/Thermo Scientific; Pittsburgh, PA)

### Quantitative real time PCR (qPCR)

Relative gene expression is shown as a ratio of the expression level of the gene of interest to that of Hypoxanthine-guanine phosphoribosyltransferase (*Hprt1*). The following primer sets were used: *Hprt1*: 5’-CCTGGTTAAGCAGTACAGCCC-3’, 5’-CAAATCCAACAAAGTCTGGCCT-3’; *Il6*: 5’-GTGGCTAAGGACCAAGACCA-3’, 5’- TAACGCACTAGGTTTGCCGA-3’; *Ifnb*: 5’- TGGG-AGATGTCCTCAACTGC-3’, 5’- CCAGGCGTAGCTGTTGTACT-3’; *Il1b*: 5’-AATTGGTCATAGCCCGCACT-3’, 5’-AAGCAATGTGCTGGTGCTTC-3’; *Mmp3*: 5’-ATGGGCCTGGAACAGTCTTG-3’, 5’-GGTTGGTACCAGTGACATCCTC-3’; *Tnf*: 5’-CACACTCACAAACCACCAAGTG-3’, 5’- ACAA-GGTACAACCCATCGGC-3’; *Bst2*: 5’-AGTTGGCAGGTCACAGTTGTT-3’, 5’- GAAGACCCAATCTGGAGCCC-3’; *Aim2* 5’-AGGCAGTGGGAACAAGACAG-3’, 5’- AAACTTCCTGACGCCACCC-3’

### Multiplex protein analysis

Serum cytokines and cytokine expression in the joints were determined by using customized V-PLEX Proinflammatory Panel 1 (Meso Scale Discovery; Rockville, MD).

### Statistical analysis

All data are presented as mean values, whereas error bars indicate SEM. Statistical tests were conducted as indicated using GraphPad Prism (GraphPad, La Jolla, CA).

## Results

### AIM2-deficiency protects from self-DNA mediated polyarthritis

**Fig 1:**
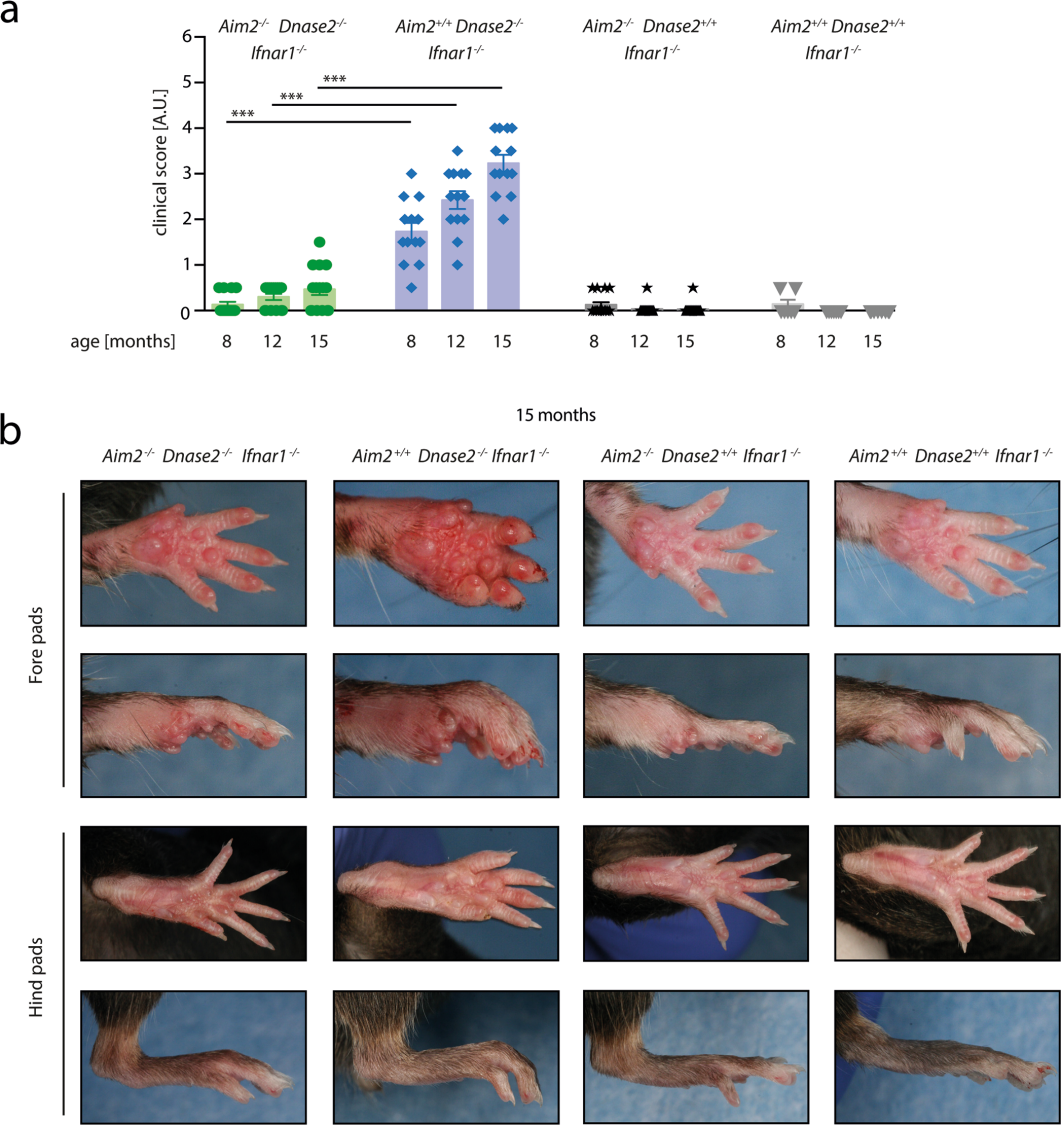
Swelling of joints in the context of *Dnase2*-deficiency is AIM2-dependent. **A:** *Aim2* ^*+/+*^ *Dnase2* ^*-/-*^ *Ifnar1* ^*-/-*^, *Aim2* ^*-/-*^ *Dnase2* ^*-/-*^ *Ifnar1* ^*-/-*^, *Aim2* ^*+/+*^ *Dnase2* ^*+/+*^ *Ifnar1* ^*-/-*^ *and Aim2* ^*-/-*^ *Dnase2* ^*+/+*^ *Ifnar1* ^*-/-*^ mice were scored for joint swelling at indicated time points in a blinded fashion. Statistical significance was assessed using a two-tailed Mann-Whitney test comparing Dnase2^*-/-*^ cohorts at 8, 12 or 15 months. **B:** Representative pictures of fore-and hind pads of Aim2^*+/+*^ Dnase2^*-/-*^ Ifnar1^*-/-*^, Aim2^*-/-*^ Dnase2^*-/-*^ Ifnar1^*-/-*^, Aim2^*+/+*^ Dnase2^*+/+*^ Ifnar1^*-/-*^ and Aim2^*-/-*^ Dnase2^*+/+*^ Ifnar1^*-/-*^ mice at the age of 15 month are shown.

To study the contribution of AIM2 to *Dnase2*-deficiency triggered chronic polyarthritis, we crossed mice deficient in *Aim2* with mice lacking *Dnase2* and the common chain 1 of the type I IFN receptor (*Aim2* ^*-/-*^, *Dnase2* ^-/-^, *Ifnar1* ^-/-^). As controls, mice deficient in *Dnase2* and *Ifnar1* (*Aim2* ^*+/+*^, *Dnase2* ^-/-^, *Ifnar1* ^-/-^), deficient in *Aim2* and *Ifnar1* (*Aim2* ^*-/-*^, *Dnase2* ^+/+^, *Ifnar1* ^-/-^) or only deficient in *Ifnar1* (*Aim2* ^*+/+*^, *Dnase2* ^+/+^, *Ifnar1* ^-/-^) were studied. For simplicity, the *Ifnar1* genotype is not specifically referred to in the following, given the fact that all mice studied were deficient for *Ifnar1*. Mice of these genotypes were housed under specific pathogen free conditions and monitored for signs of polyarthritis. Consistent clinical signs of polyarthritis were observed around 8 months after birth. As such, *Dnase2*-deficient mice being wild type for *Aim2* displayed a significant mean arthritis score of 1.8 compared to *Dnase2*-competent mice (Fig 1A). In the additional absence of *Aim2*, *Dnase2*-deficient mice (*Aim2* ^*-/-*^ *x Dnase2* ^-/-^) showed a significant reduction in their signs of arthritis with a clinical score that was comparable to *Dnase2*-competent mice. With increasing age, signs of chronic polyarthritis progressed to a score of up to 3.4 in the *Aim2* ^*+/+*^ *x Dnase2* ^-/-^ cohort at 15 months, whereas *Aim2* ^*-/-*^ *x Dnase2* ^-/-^ mice showed greatly reduced signs of arthritis (mean score of 0.5). Of note, in line with previous reports, arthritis was more pronounced in fore pads than in hind pads (Fig 1B). Altogether, these results indicated that AIM2 is required for the development of polyarthritis in the context of *DNase2*-deficiency.

### AIM2-deficiency blunts pro-inflammatory as well as antiviral gene expression in diseased joints

**Fig 2:**
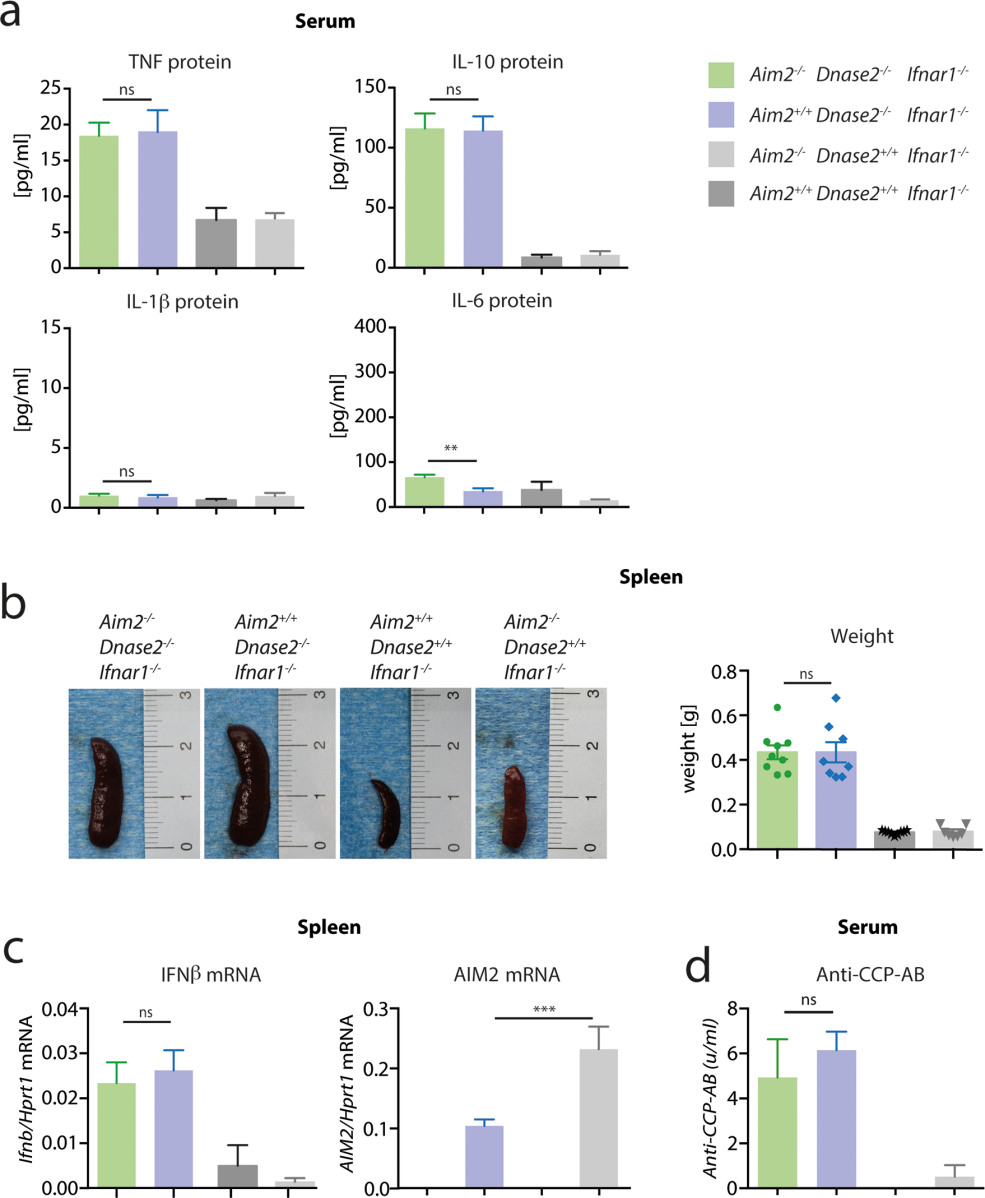
Systemic proinflammatory status in *Dnase2* ^*-/-*^ mice is independent of AIM2. **A:** Serum was collected from *Aim2* ^*+/+*^ *Dnase2* ^*-/-*^ *Ifnar1* ^*-/-*^, *Aim2* ^*-/-*^ *Dnase2* ^*-/-*^ *Ifnar1* ^*-/-*^, *Aim2* ^*+/+*^ *Dnase2* ^*+/+*^ *Ifnar1* ^*-/-*^ *and Aim2* ^*-/-*^ *Dnase2* ^*+/+*^ *Ifnar1* ^*-/-*^ mice at the age of 15 month and analyzed for the depicted cytokines. **B:** Spleen weight and representative pictures of spleens from mice at the age of 15 month. **C:** Relative IFNβ gene expression in spleen tissue normalized to the expression level of HPRT1 is shown. **D:** Anti-cyclic citrullinated peptide antibody (Anti-CCP-AB) was measured in serum samples as in (**A**). Data are presented as mean values + SEM, whereas statistical significance was assessed using a two-tailed, unpaired t-test comparing the *Dnase2* ^*-/-*^ cohorts.

As mentioned above, it has been proposed that joint inflammation in this mouse model is driven by systemic inflammation. Therefore, to elucidate the mechanism of *Aim2*-deficiency ameliorating *DNase2* ^*-/-*^-associated polyarthritis, we next assessed systemic signs of inflammation in these mice. *Dnase2*-deficient mice showed markedly increased levels of TNF and IL-10 in serum, however, this was observed irrespective of their *Aim2* genotype (Fig 2A). Surprisingly, levels of the disease-relevant cytokines IL-6 and IL-1β were not elevated in serum of *Dnase2* ^*-/-*^ animals (Fig 2A) and cytokines associated with increased activation of the adaptive branch of the immune system (e.g. IL-2, IL-4 or IFNγ) were also not significantly increased above background level (data not shown). *Dnase2*-deficient mice developed marked splenomegaly, with spleen sizes being 4–5 fold increased compared to control animals (Fig 2B). However, as observed for serum levels of TNF and IL-10, the absence of AIM2 had no impact on this phenomenon. Spleen enlargement was associated with an increased expression of IFNβ in splenic tissue (Fig 2C), again being independent of the *Aim2* genotype. Moreover, anti-cyclic citrullinated peptide antibodies were also observed in *Dnase2*-deficient mice irrespective of their *Aim2* genotype (Fig 2D).

**Fig 3:**
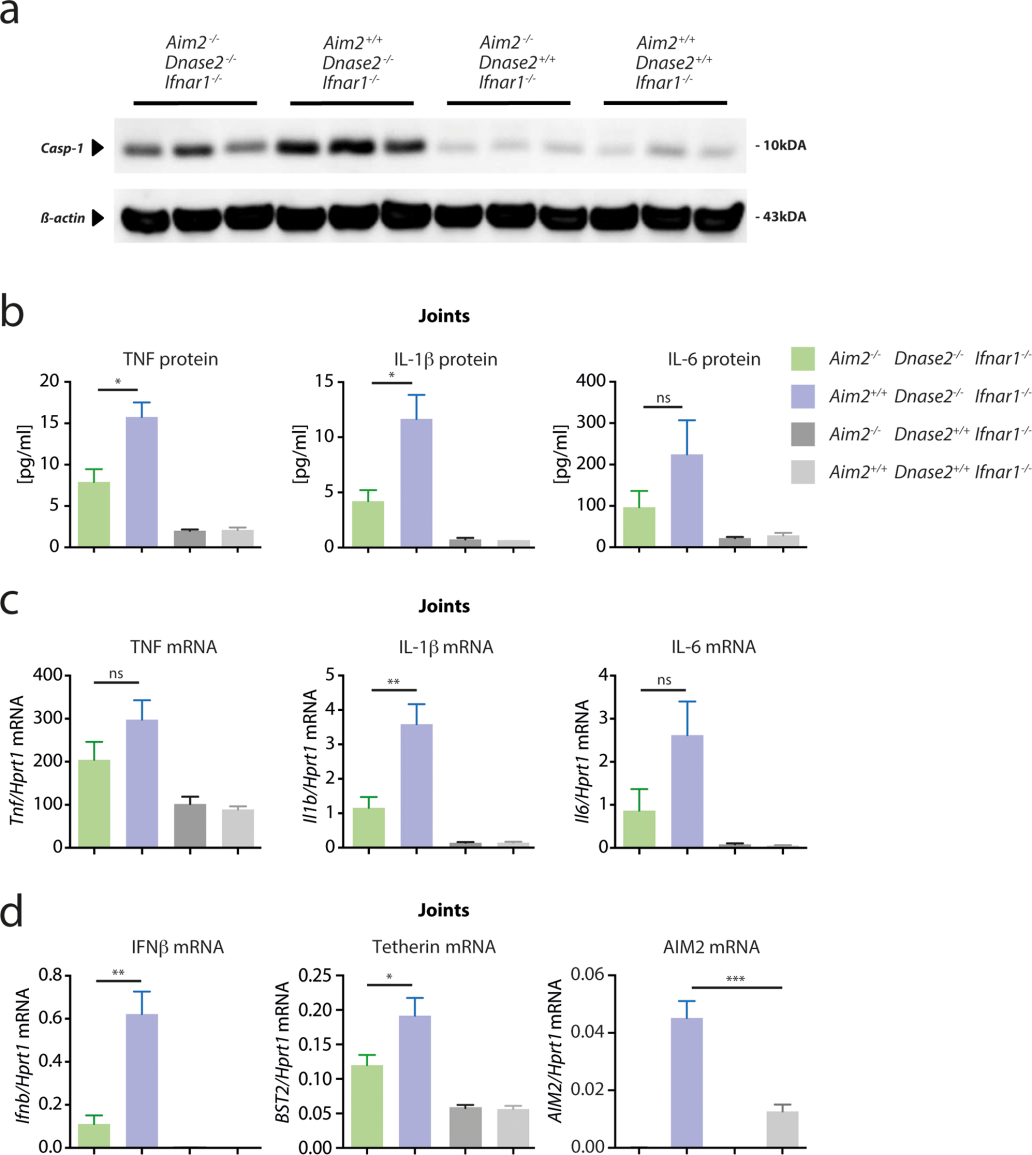
Gene expression of proinflammatory cytokines in joints of Dnase2 deficient mice relies on AIM2. **A:** Protein lysates were generated from the joints of 15 months old mice and immunoblotted for the presence of cleaved Caspase-1, whereas β-Actin served as a loading control. Three independent protein lysates were analyzed per cohort. **B:** Cytokine levels of lysates as in (**A**) were determined. **C** and **D:** qPCR analysis of joints of the four different cohorts is shown for the indicated transcripts. The mRNA levels for the indicated cytokines are expressed relative to mRNA of HPRT1. Data are presented as mean values + SEM, whereas statistical significance was assessed using a two-tailed, unpaired t-test comparing the *Dnase2* ^*-/-*^ cohorts.

While these data could not explain the positive impact of *Aim2*-deficiency in this mouse model, analyzing caspase-1 activation and cytokine expression in joints of *Dnase2*-deficient mice revealed a different picture. Compared to *Dnase2*-competent mice, joints of *Aim2* ^*+/+*^ *x Dnase2* ^-/-^ mice showed a marked increase in cleaved caspase-1, which was largely reduced in the absence of AIM2 (Fig 3A). Concomitant with this increased inflammasome activation, the expression of pro-inflammatory cytokines such as TNF, IL-6 and IL-1β expression was strongly increased both at protein, as well as at the mRNA level in joints of *Dnase2*-deficient mice (Fig 3B and 3C). Of note, the expression of these pro-inflammatory cytokines was reduced in the absence of *Aim2*. At the same time, diseased joints also displayed a robust induction of IFNβ and also interferon stimulated gens such as *Aim2*, *Bst2* or Viperin were upregulated at the mRNA or protein level (Fig 3D and S2 Fig). This phenomenon can be explained by the fact that these genes are also directly induced upon cytosolic PRR stimulation, independently of the IFNAR loop [24]. Interestingly, the expression of these ISGs was also *Aim2*-dependent, most prominently IFNβ.

### AIM2 governs macrophage infiltration and local joint destruction in *Dnase2*-deficient mice

**Fig 4:**
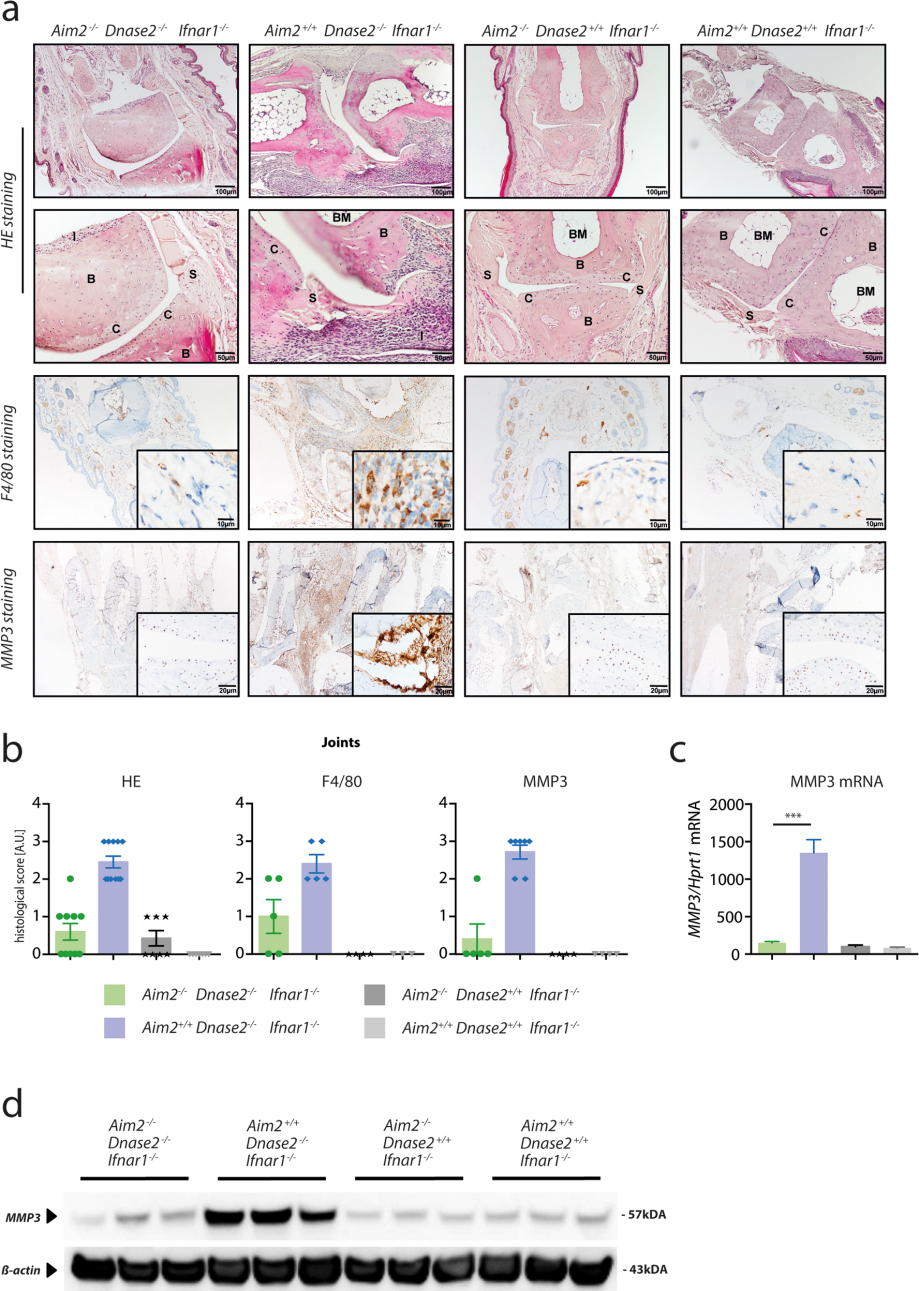
Local inflammation of joints in Dnase2 deficient mice is largely AIM2 dependent. **A:** Joint sections of 15 month old mice of the depicted genotypes were stained with haematoxylin/eosin, F4/80 and MMP3. B, Bone; BM, Bone marrow; C, cartilage; I, Infiltrate; S, Synovitis. **B:** Histological scores of joint sections from 15 month old mice stained with HE, F4/80 or MMP3: 0, no overt signs of synovitis / infiltration; 1, low grade synovitis / infiltration; 2, intermediate grade synovitis / infiltration; 3, high grade synovitis / infiltration. **C:** RNAs were isolated from the joints of 15 months old mice to quantify MMP3 expression normalized to HPRT1. Data are presented as mean values + SEM, whereas statistical significance was assessed using a two-tailed, unpaired t-test comparing the *Dnase2* ^*-/-*^ cohorts. **D**: Protein lysates were generated from the joints of 15 months old mice and immunoblotted for the presence of MMP3, whereas β-Actin served as a loading control. Three independent protein lysates were analyzed per cohort. Data are presented as mean values + SEM, whereas statistical significance was assessed using a two-tailed Mann-Whitney test (**b**) or using a two-tailed, unpaired t-test (**c**) comparing the *Dnase2* ^*-/-*^ cohorts.

At 15 months of age, joints of *Aim2* ^*+/+*^ *x Dnase2* ^-/-^ mice showed severe signs of synovitis with hyperproliferation of synovial cells and massive immune cell infiltration, associated with pannus formation, cartilage destruction and bone erosion (Fig 4A and 4B). The immune cell infiltrate, most prominently pannus formation, was mainly dominated by the presence of macrophages, as revealed by F4/80 immunohistochemistry. Moreover, as a quantitative measure of local catabolic activity, expression of matrix metalloproteinase-3 (*Mmp3*) was strongly induced in joints of *Aim2* ^*+/+*^ *x Dnase2* ^-/-^ animals as observed by immunohistochemistry (Fig 4A and 4B) and also by qPCR and immunoblotting of joint sections (Fig 4C and 4D). In line with the arthritis scores and the pro-inflammatory cytokine profile, histological signs of arthritis, macrophage infiltration and *Mmp3* expression were largely decreased in the absence of *Aim2*.

## Discussion

Altogether our data show that AIM2 plays an important role in the recognition of endogenous DNA species in the context of *Dnase2* ^*-/-*^ associated arthritis, thereby establishing AIM2 as a DAMP sensing PRR. Interestingly, the impact of AIM2 deficiency was primarily observed in diseased joints and not seen at the systemic level (e.g. splenomegaly or anti-CCP antibodies). These data imply that joint inflammation and subsequent tissue destruction are primary effects and mainly governed by local disease mechanisms and not just secondary to a systemic elevation of pro-inflammatory cytokines. Moreover these results suggest that AIM2 activation drives disease progression in a tissue specific manner in this model. This concept is in line with a previous study that has shown a predominant accumulation of self-DNA in joint tissue of *Dnase2*-deficient mice, thereby arguing for an increase in ligand availability at the site of inflammation [21]. At the same time, it was also demonstrated that *Dnase2*-deficieny not only results in DNA accumulation resulting from undigested, phagocytized material, but also from cell-autonomous sources [21]. As such, damaged DNA expelled from nuclei can gain access to the cytosol, yet is subject to autophagy-mediated transport into DNase II containing lysosomes under normal conditions. The fact that cell autonomous and phagocytized material can both serve as ligands in the context of *Dnase2*-deficiency might explain why ablating AIM2 does not ameliorate all aspects of this disease model. Of note, with AIM2 being largely confined to the myeloid lineage, it is expected that AIM2 has no impact on self-DNA recognition in non-myeloid cells.

As observed by caspase-1 immunoblotting, *Dnase2*-deficient mice displayed signs of inflammasome activation in diseased joints, and consistent with its role as an inflammasome receptor, *Aim2*-deficiency led to a decreased activation of caspase-1. In line with blunted inflammasome activation, IL-1β levels were decreased in joints of *Aim2* ^*-/-*^ *x Dnase2* ^-/-^ mice, which could explain part of the pathomechanism being operational in this disease model [20]. Interestingly, beyond its impact on the inflammasome, *Aim2* deficiency also affected the expression of pro-inflammatory and antiviral genes in diseased joints, a phenomenon that had previously been ascribed to STING in this mouse model [17]. For example, expression of IFNβ, a cytokine that is completely STING-dependent in the context of cytosolic DNA recognition [25], was greatly reduced in diseased joints of AIM2-deficient mice. Whereas IFNβ itself does not play a role in this disease model (global *Ifnar1* deficiency), these results indicate that the signaling cascade leading to IFNβ production is indeed operational. This is also documented by the fact that IFN-stimulated genes are upregualted in joints of *Dnase2*-deficient mice. While these results could imply an epistatic relationship in signaling, with AIM2 functioning upstream of STING, we hypothesize that this is not the case. Most importantly, *in vitro* AIM2-deficient macrophages display normal pro-inflammatory gene expression and even increased type I IFN production upon cytosolic DNA delivery, while caspase-1 dependent processing of pro-IL-1β and pro-IL-18 and pyroptosis are abrogated [9, 26, 27]. These observations clearly argue against a cell-autonomous role of AIM2 in this model functioning upstream or in conjunction with STING signaling. Moreover, the fact that *Aim2* deficiency differentially impacts on pro-inflammatory cytokine expression in joints and in spleen furthermore disfavors a cell intrinsic role of AIM2 positively regulating STING-mediated signal transduction. In fact we speculate that the AIM2-dependent processing of IL-1 family cytokines and pyroptosis-dependent release of DAMPs leads to the priming of neighboring cells within joint tissue. A similar scenario has recently been proposed in the context of intramuscular DNA vaccination, where *Aim2*-deficient animals showed greatly reduced expression of pro-inflammatory and antiviral cytokines at the sight of DNA application [28]. In this context, it is furthermore noteworthy that pro-inflammatory cytokines can dramatically enhance the responsiveness of non-myeloid cells to DNA stimulation via the STING axis [29]. Extrapolated to the in vivo situation, this would imply that AIM2 functions in a non-cell autonomous fashion to trigger inflammation, with AIM2 not being active in the cells that express pro-inflammatory cytokines. Nevertheless, it has also been reported that AIM2, when stimulated at lower ligand concentrations, can engage signaling cascades other than the canonical inflammasome pathway. To this end, it was shown that AIM2 can recruit procaspase-8 in an ASC-dependent fashion at ligand concentrations below the threshold of caspase-1 activation or in the absence of caspase-1 respectively [30, 31]. While not formally established, caspase-8 recruitment by AIM2 could drive NF-KB activation and as such impact on pro-inflammatory gene expression under these conditions. Altogether, the exact mechanism of AIM2 contributing to pro-inflammatory and antiviral gene expression in this model remains to be determined, while it should be informative to dissect the contribution of different cells types in the *in vivo* situation.

With the predominant involvement of the adaptive branch of the immune system unequivocally being documented in rheumatoid arthritis, the relevance of erroneous DNA sensing by PRRs has yet to be established in this disease entity. However, the *Dnase2* ^-/-^ model shares a number of prominent features with Systemic juvenile idiopathic arthritis (sJIA), which can be considered an autoinflammatory disease [20, 32]. Having identified AIM2 as a critical pro-inflammatory driver in this disease model provides a molecular target that could be of interest for future therapeutic intervention.

**Note added in proof**: While this manuscript was under review, Ellen Gravallese and colleagues have published a likewise approach of studying the role of AIM2 in the context of *Dnase2* ^*-/-*^ x *Ifnar1* ^*-/-*^ induced polyarthritis [33]. They also found a critical role for AIM2 in driving disease pathology (clinical arthritis score, pro-inflammatory cytokine production in joints), yet with a slightly lower impact compared to our study. Moreover, in their analysis *Aim2* deficiency led to a considerable decrease in IL-18 levels in serum and in joint tissue, a phenomenon that we did not observe in our cohorts (data not shown). It is possible that differences in mouse strains used or housing conditions account for these discrepancies. However, given the fact that IL-18 deficiency does not protect *Dnase2*-deficient animals from polyarthritis or pro-inflammatory gene expression [20], *Aim2*-dependent IL-18 production or maturation appears to be an epiphenomenon rather than a cause in this disease model. Indeed, our data suggest that *Aim2* deficiency dampens caspase-1 activation and associated IL-1β secretion and that it also impacts on pro-inflammatory cytokine expression in joint tissues.

## Supporting Information

S1 Fig Generation of *Aim2*-deficient mice. **A:** The gene targeting approach that was taken to generate *Aim2* ^-/-^ mice is depicted. **B:** Representative genotyping result for wildtype, *Aim2* ^-/-^ and *Aim2* ^*+*/-^ mice (Primer binding positions are depicted in **A**). **C**: LPS- primed bone marrow derived macrophages of wildtype or *Aim2* ^-/-^ were stimulated with the indicated stimuli and analyzed for IL-1β production 6 hours after stimulation.
(PDF)

S2 Fig Viperin expression in joints of *Dnase2*-deficient mice. Protein lysates were generated from the joints of 15 months old mice and immunoblotted for the presence of Viperin, whereas β-Actin served as a loading control. Three independent protein lysates were analyzed per cohort.
(PDF)
