# Correction: AIM2 Drives Joint Inflammation in a Self-DNA Triggered Model of Chronic Polyarthritis

**DOI:** 10.1371/journal.pone.0202364

**Published:** 2018-08-09

**Authors:** Christopher Jakobs, Sven Perner, Veit Hornung

In Fig 1 panel A, the data sets of the control groups (Aim2^-/-^ Dnase2^+/+^ Ifnar1^-/-^ and Aim2^+/+^ Dnase2^+/+^ Ifnar1^-/-^) are shown as mirror images of the original data. In Fig 2 panel A, the shades of grey in the legend (Aim2^-/-^ Dnase2^+/+^ Ifnar1^-/-^ and Aim2^+/+^ Dnase2^+/+^ Ifnar1^-/-^) are swapped. In Fig 2 panel B the third and fourth labels are swapped. Please see the correct Figs 1 and 2 here.

**Fig 1:**
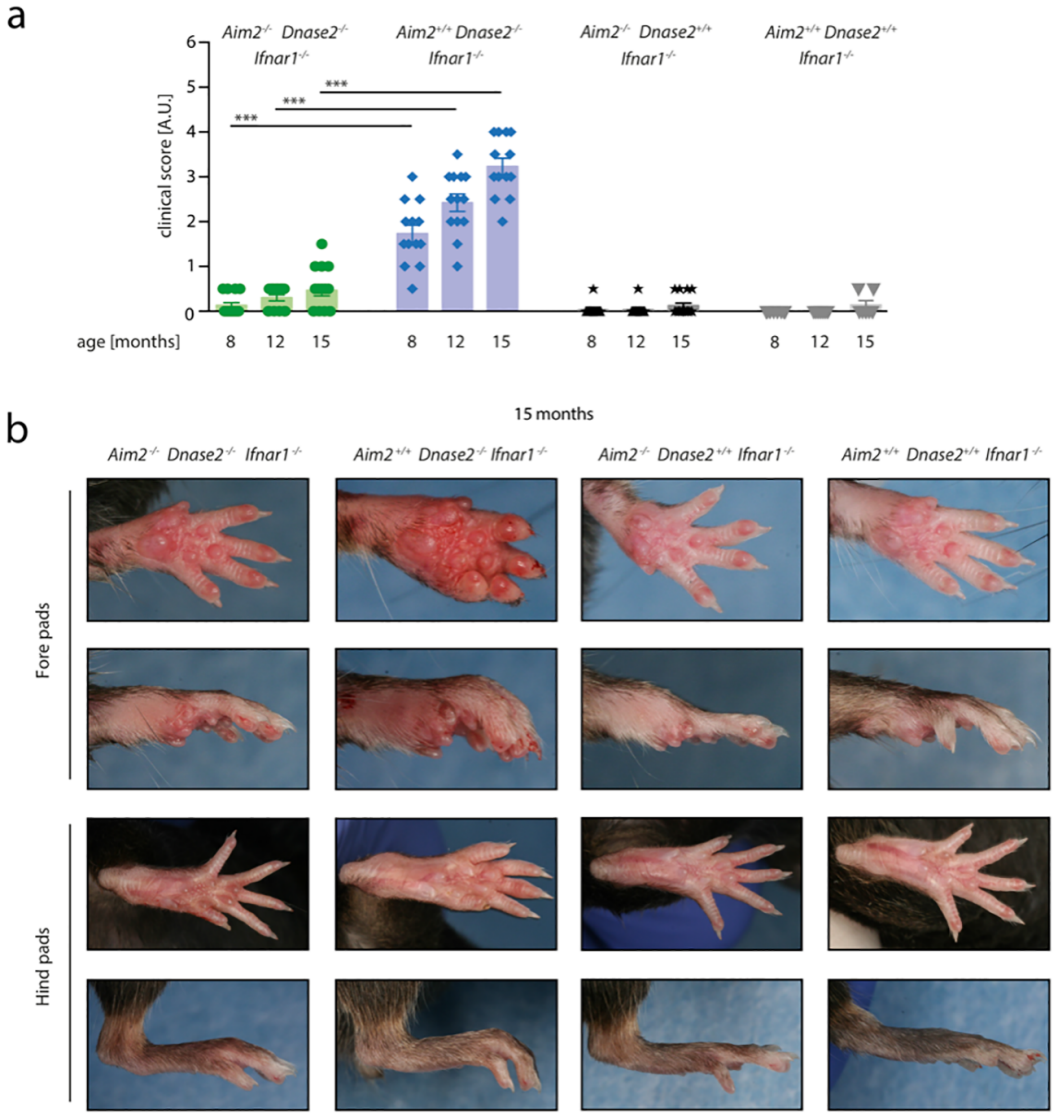
Swelling of joints in the context of *Dnase2*-deficiency is AIM2-dependent. A: *Aim2*^*+/+*^ *Dnase2*^*-/-*^ *Ifnar1*^*-/-*^, *Aim2*^*-/-*^ *Dnase2*^*-/-*^ *Ifnar1*^*-/-*^, *Aim2*^*+/+*^ *Dnase2*^*+/+*^ *Ifnar1*^*-/-*^ *and Aim2*^*-/-*^ *Dnase2*^*+/+*^ *Ifnar1*^*-/-*^ mice were scored for joint swelling at indicated time points in a blinded fashion. Statistical significance was assessed using a two-tailed Mann-Whitney test comparing Dnase2^*-/-*^ cohorts at 8, 12 or 15 months. B: Representative pictures of fore-and hind pads of Aim2^*+/+*^ Dnase2^*-/-*^ Ifnar1^*-/-*^, Aim2^*-/-*^ Dnase2^*-/-*^ Ifnar1^*-/-*^, Aim2^*+/+*^ Dnase2^*+/+*^ Ifnar1^*-/-*^ and Aim2^*-/-*^ Dnase2^*+/+*^ Ifnar1^*-/-*^ mice at the age of 15 month are shown.

**Fig 2:**
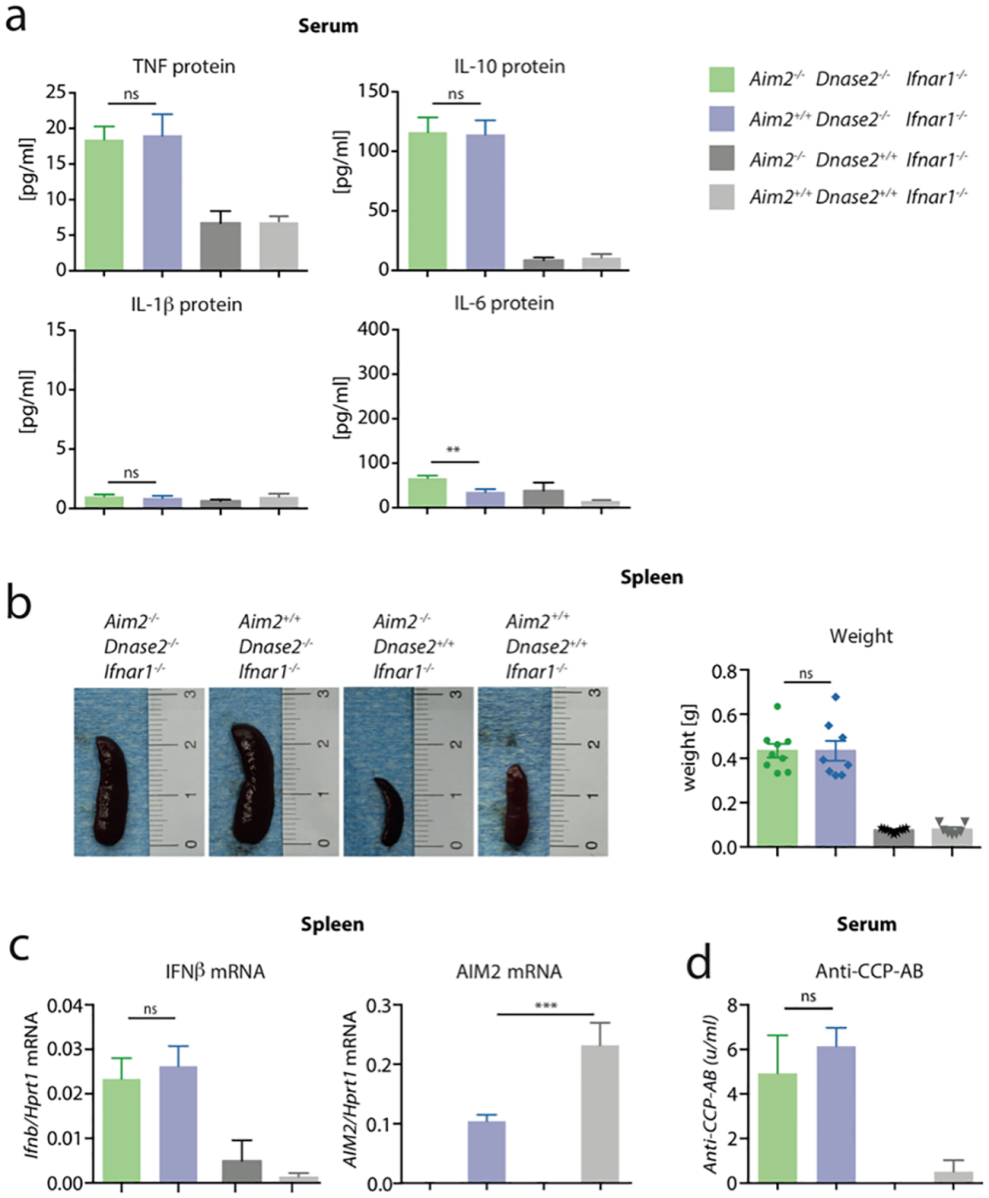
Systemic proinflammatory status in *Dnase2*^*-/-*^ mice is independent of AIM2. A: Serum was collected from *Aim2*^*+/+*^ *Dnase2*^*-/-*^ *Ifnar1*^*-/-*^, *Aim2*^*-/-*^ *Dnase2*^*-/-*^ *Ifnar1*^*-/-*^, *Aim2*^*+/+*^ *Dnase2*^*+/+*^ *Ifnar1*^*-/-*^ *and Aim2*^*-/-*^ *Dnase2*^*+/+*^ *Ifnar1*^*-/-*^ mice at the age of 15 month and analyzed for the depicted cytokines. B: Spleen weight and representative pictures of spleens from mice at the age of 15 month. C: Relative IFNβ gene expression in spleen tissue normalized to the expression level of HPRT1 is shown. D: Anti-cyclic citrullinated peptide antibody (Anti-CCP-AB) was measured in serum samples as in (A). Data are presented as mean values + SEM, whereas statistical significance was assessed using a two-tailed, unpaired t-test comparing the *Dnase2*^*-/-*^ cohorts.
