# Supplementary material for: AIM2 Drives Joint Inflammation in a Self-DNA Triggered Model of Chronic Polyarthritis
Source: PLoS One. 2015 Jun 26;10(6):e0131702. doi: 10.1371/journal.pone.0131702 (PMC4482750; doi:10.1371/journal.pone.0131702)
Supplement: S1 Fig — A: The gene targeting approach that was taken to generate Aim2 -/- mice is depicted. B: Representative genotyping result for wildtype, Aim2 -/- and Aim2 +/- mice (Primer binding positions are depicted in A). C: LPS- primed bone marrow derived macrophages of wildtype or Aim2 -/- were stimulated with the indicated stimuli and analyzed for IL-1β production 6 hours after stimulation. (PDF) [file pone.0131702.s001.pdf]

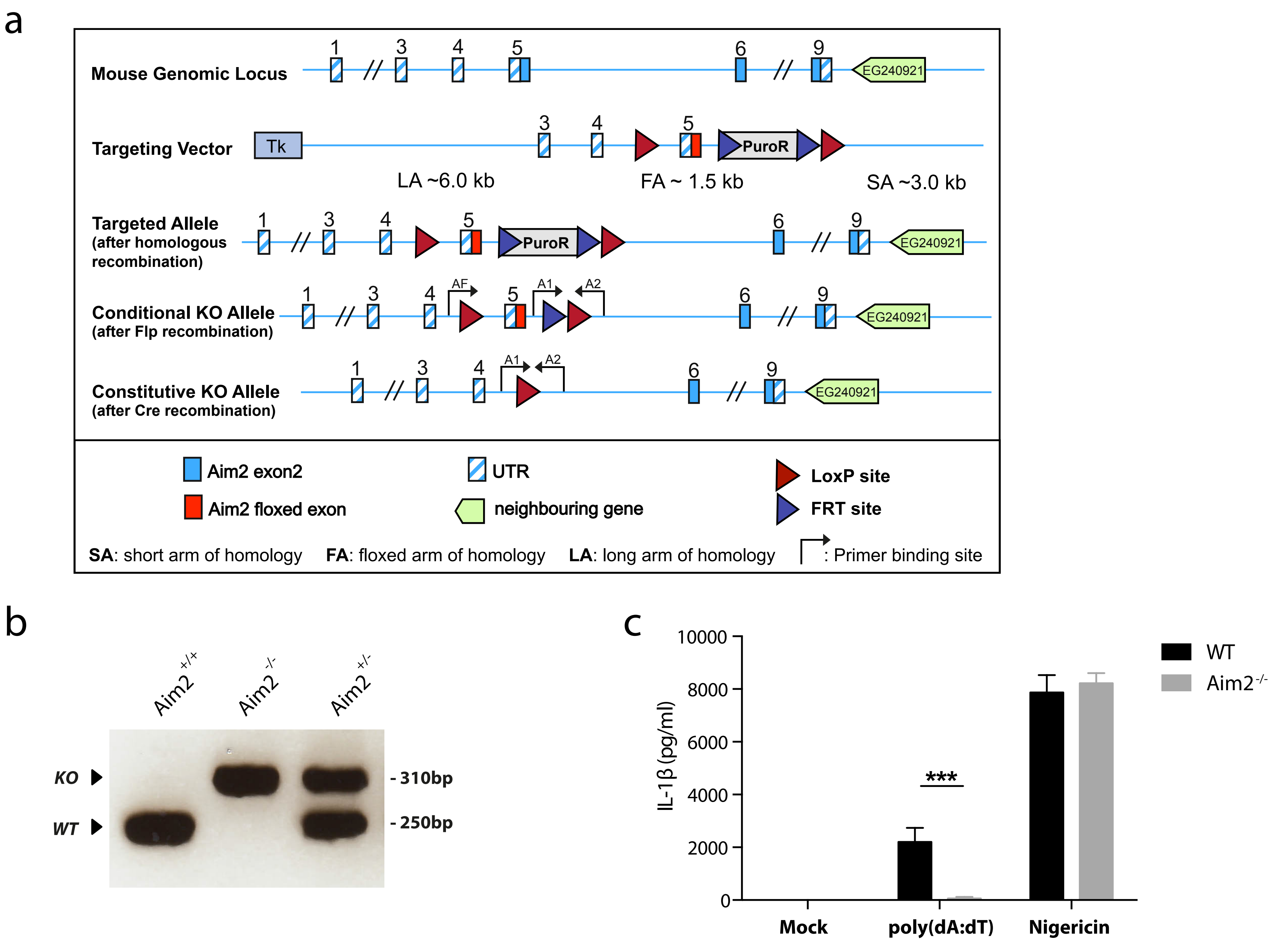

Figure S1. Generation of Aim2-deficient mice

A: The gene targeting approach that was taken to generate Aim2$^{-/-}$ mice is depicted. **B:** Representative genotyping result for wildtype, Aim2$^{-/-}$ and Aim2$^{+/-}$ mice (Primer binding positions are depicted in **A**). **C:** LPS-primed bone marrow derived macrophages of wildtype or Aim2$^{-/-}$ were stimulated with the indicated stimuli and analyzed for IL-1β production 6 hours after stimulation.
