# Supplementary material for: AIM2 Drives Joint Inflammation in a Self-DNA Triggered Model of Chronic Polyarthritis
Source: PLoS One. 2015 Jun 26;10(6):e0131702. doi: 10.1371/journal.pone.0131702 (PMC4482750; doi:10.1371/journal.pone.0131702)
Supplement: S2 Fig — Protein lysates were generated from the joints of 15 months old mice and immunoblotted for the presence of Viperin, whereas β-Actin served as a loading control. Three independent protein lysates were analyzed per cohort. (PDF) [file pone.0131702.s002.pdf]

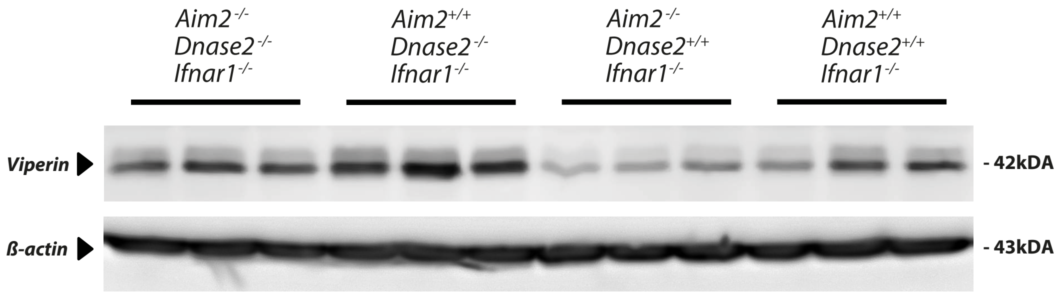

Figure S2. Viperin expression in joints of *Dnase2*-deficient mice.
Proteins lysates were generated from the joints of 15 months old mice and immunoblotted for the presence of Viperin, whereas β-Actin served as a loading control. Three independent protein lysates were analyzed per cohort.
